# Associations between open drain flooding and pediatric enteric infections in the MAL-ED cohort in a low-income, urban neighborhood in Vellore, India

**DOI:** 10.1186/s12889-019-7268-1

**Published:** 2019-07-10

**Authors:** David M. Berendes, Juan S. Leon, Amy E. Kirby, Julie A. Clennon, Suraja J. Raj, Habib Yakubu, Katharine A. Robb, Arun Kartikeyan, Priya Hemavathy, Annai Gunasekaran, Sheela Roy, Ben Chirag Ghale, J. Senthil Kumar, Venkata Raghava Mohan, Gagandeep Kang, Christine L. Moe

**Affiliations:** 1 Department of Environmental Health, Rollins School of Public Health, Emory University, Atlanta, GA USA; 2 Center for Global Safe Water, Sanitation, and Hygiene, Rollins School of Public Health, Emory University, Atlanta, GA USA; 3 Hubert Department of Global Health, Rollins School of Public Health, Emory University, Atlanta, GA USA; 4 Department of Biostatistics, Rollins School of Public Health, Emory University, Atlanta, GA USA; 5 Wellcome Research Laboratory, Christian Medical College, Vellore, India; 6 Department of Community Health, Christian Medical College, Vellore, India; 7 Present address: Waterborne Disease Prevention Branch, Division of Foodborne, Waterborne, and Environmental Diseases, Centers for Disease Control and Prevention, Atlanta, GA USA

**Keywords:** Open drains, Flooding, Enteric infections, Urban infrastructure, Pediatric health, Environmental contamination

## Abstract

**Background:**

Open drains are common methods of transporting solid waste and excreta in low-income urban neighborhoods. Open drains can overflow due to blockages with solid waste and during rainfall, posing exposure risks. The goal of this study was to evaluate whether pediatric enteric infection was associated with open drains and flooding in a dense, low-income, urban neighborhood.

**Methods:**

As part of the MAL-ED study in Vellore, India, a cohort of 230 children provided stool specimens at 14–17 scheduled home visits and during diarrheal episodes in the first two years of life. All specimens were analyzed for enteric pathogens. Caregivers in 100 households reported on flooding of drains and households and monthly frequency of contact with open drains and flood water. Household GPS points were collected. Monthly rainfall totals for the Vellore district were collected from the Indian Meteorological Department. Clustering of reported drain and house flooding were identified by Kulldorff’s Bernoulli Spatial Scan. Differences in enteric infection were assessed for household responses and spatial clusters, with interactions between reported flooding and rainfall to approximate monthly drain flooding retrospectively, using multivariable, mixed-effects logistic regression models.

**Results:**

Coverage of household toilets was low (33%), and most toilets (82%) discharged directly into open drains, suggesting poor neighborhood fecal sludge management. Odds of enteric infection increased significantly with total monthly rainfall for children who lived in households that reported that the nearby drain flooded (4% increase per cm of rain: OR: 1.04, 95% CI: 1.00–1.08) and for children in households in a downstream spatial cluster of reported drain flooding (5% increase per cm of rain: OR: 1.05, 95% CI: 1.01–1.09). There was no association between odds of enteric infection and frequency of reported contact with drain or floodwater.

**Conclusions:**

Children in areas susceptible to open drain flooding had increased odds of enteric infection as rainfall increased. Results suggested that infection increased with rainfall due to neighborhood infrastructure (including poor fecal sludge management) and not frequency of contact. Thus, these exposures may not be mitigated by changes in personal behaviors alone. These results underscore the importance of improving the neighborhood environment to improve children’s health in low-income, urban settings.

**Electronic supplementary material:**

The online version of this article (10.1186/s12889-019-7268-1) contains supplementary material, which is available to authorized users.

## Background

Enteric infections may be transmitted through contaminated water, poor sanitation, and inadequate hygiene (WASH). While research on the impact of WASH on reported diarrheal symptoms is common [1], research on the impact of WASH on both symptomatic and asymptomatic infections is rare [2]. Furthermore, there is increasing recognition of the contributions of both symptomatic and asymptomatic pediatric enteric infections to poor child health, growth, and cognition [3–6]. Most previous studies of WASH—and especially sanitation—have focused on rural environments; however, there is a critical need to understand the complex relationship between WASH and enteric infections in the world’s rapidly expanding urban environments, which already include over half of the global population [7, 8].

Urban infrastructure can mitigate or facilitate personal exposures to fecal contamination in the household and public environments depending on the effectiveness of the fecal sludge management, drainage, and wastewater systems [8–14]. While toilet ownership alone has been shown to reduce certain types of enteric infections [15], safely-managed sanitation systems that include functional household or public toilets and accompanying safe fecal sludge management (FSM) along the entire sanitation chain are necessary to contain excreta and prevent human contact in both the household and neighborhood environments [16–18]. In the absence of safely-managed sanitation, dense urban environments can pose risks to residents both near and downstream of fecal discharge locations because of poor maintenance and cleaning of sanitation facilities and uncontained FSM infrastructure, like open drains [9–12, 17, 19–29]. Direct contact with open drain water, or associated floodwaters, is common for children in low-income settings [9, 10]. Drain water has high levels of fecal contamination because the high costs and challenging logistics associated with implementing sewerage, or emptying pit latrines or septic tanks, make open drains a common fate for untreated excreta [9, 10, 17, 30–36]. While multiple, recent quantitative microbial risk assessments (QMRAs) in sub-Saharan Africa, West Africa, and Europe have suggested that exposure to open drains and drain flooding may be important risk factors for pediatric enteric infections [9–12, 37], no observational studies have confirmed these risks.

Children’s exposure to environmental fecal contamination may vary by magnitude of the contamination in the environment, the frequency and type of contact (direct or indirect), and the ingested dose. For example, direct contact with highly contaminated open drains can result in incidental ingestion of a high dose of fecal contamination from a single event, while consumption of municipal water may yield the same cumulative dose through more frequent exposures to lower concentrations of fecal contamination [9, 10, 37, 38]. Rainfall may interact with urban infrastructure in numerous ways to cause flooding of open drains. By itself, rainfall—especially heavy rainfall events, may represent a significant risk factor for enteric bacterial infections [39]. In urban environments, rainfall may dilute the concentrations of pathogens in open drain water but make them more mobile. Often, the dose of pathogens ingested after a single contact with flood water may still be high enough to cause infection [37, 40, 41]. The interactions of rainfall and movement of fecal waste is still the subject of intense research, including development of systems-level models to understand complex interactions with the built environment [42].

Open drains are commonly used to transport runoff and fecal sludge from households and have been recognized, along with their associated flooding, as risk factors for infection in QMRAs [9–12]. Yet, no studies have attempted to measure adverse health outcomes associated with these complex urban environmental transmission pathways. The goal of this study was to evaluate whether pediatric enteric infection is associated with either 1) reported contact with, or 2) reported flooding of, open drains in a low-income, urban neighborhood of Vellore, India, as measured by identification of households or areas with reported drain flooding and retrospective aggregation of rainfall data. We hypothesized that children living in households or areas that reported open drain flooding would have increased enteric infections as rainfall increased. Separately, we hypothesized that increases in reported contact with open drains would be associated with increases in enteric infection. This study examined infrastructural and behavioral factors separately to better understand how the associations between exposures to fecal contamination in the public domain and pediatric enteric infections vary in low-income, urban settings.

## Methods

### Data sources

This study was a sub-analysis of several studies conducted in the Old Town neighborhood of Vellore, India. It used three sources of data:

1. Data on enteric infection (the main outcome of this study) and household socioeconomic status (an a priori covariate for adjusted models in this study) from The Etiology, Risk Factors, and Interactions of Enteric Infections and Malnutrition and Consequences for Child Health and Development Project (MAL-ED study);
2. Exposure data (reported habitual flooding of open drains and frequency of contact with open drains: the main exposure variables in this study) from the SaniPath Exposure Assessment Tool;
3. Publicly-available district-level data on cumulative monthly rainfall and average monthly temperature (additional covariates for adjusted models in this study) from the Customized Rainfall Information System (CRIS) Hydromet Division of the India Meteorological Department [6, 43–47] and World Weather Online [48].

The MAL-ED study in Vellore was conducted by the Christian Medical College and Hospital, Vellore (CMC). The birth cohort was enrolled from March 2010 – February 2012, and data collection ended in February 2014. Community mapping and household surveys as part of the SaniPath Exposure Assessment Tool deployment were conducted by Emory University, in collaboration with CMC, in February – March 2014. Thus, assessment of exposures (household conditions, reported drain flooding, and reported contact with drain and flood waters) occurred after outcome measurement. Some of the methods of this study (enteric infection measurement and exposure data collection) have been published previously [15].

### Study site

Annually, Vellore has a dry season (January – May), a southwest monsoon (June – September), and a northeast monsoon (October – December) [43]. Old Town is a small, low-income urban neighborhood with high population density (approximately 42,000/km^2^), poor sanitation, and high burden of enteric disease [28, 43]. CMC has a longstanding relationship with the community, including mapping of neighborhood infrastructure (e.g. open drains) in previous studies [43].

### Ethical approval

Ethical approval for the MAL-ED study in Vellore was obtained from the CMC Institutional Review Board (IRB) prior to subject recruitment [43]. Approval was also obtained from the Emory University IRB and the CMC IRB prior to the SaniPath exposure assessment tool deployment. Informed consent was obtained onsite from the child’s caregiver prior to survey administration.

### Measurement of enteric infection (MAL-ED study)

Stool specimens were collected from one study child per household as described in the MAL-ED study protocols [43, 49] and in a previous study [15]. Briefly, specimens were collected monthly over the child’s first year of life, and then every 2–3 months over the next year (“routine stool”). Caregivers also submitted specimens at each diarrheal event during the study period (“diarrheal stool”). All specimens were tested for multiple enteric pathogens: 8 bacteria, 9 helminths, 9 protozoa, and 4 viruses (organisms listed in the Additional file 1 by culture, microscopy, immunoassay, and PCR as described previously [4, 50].

### Assessment of open drain contact and flooding (SaniPath exposure assessment tool)

Methods for the SaniPath Exposure Assessment Tool have been previously described [15]. Transect walks with a community leader identified public locations of potential fecal exposures, including public toilets and animal grazing areas, which were documented using Global Positioning System (GPS) points collected on Garmin eTrex Venture HC devices (Garmin International Inc., Olathe, Kansas, USA, accurate to < 10 m). Locations of open drains (including direction of flow) and the primary open defecation field were previously mapped by CMC staff [43].

Household selection has been previously described in detail [28]. Briefly, 100 households were surveyed (standardized a priori in the SaniPath Exposure Assessment Tool [44]), 25 of which were selected based on the results of a hygiene survey [51] (completed prior to the SaniPath Tool deployment) to compare fecal contamination levels and enteric pathogens in a previously described cross-sectional sub-study [28]. The other 75 households were chosen randomly from a list of all households in the MAL-ED study area obtained from CMC [43].

Household surveys assessed the following: 1) flooding of the drain near the household (respondent answered yes/no as to whether the drain ever flows over its borders) and house (respondent answered yes/no as to whether floodwater ever enters the home) during/after rainfall; 2) presence of a toilet (as well as where the toilet discharged to: septic tank, drain, or other); and 3) the average frequency of contact with drains and floodwater by the study child (per month). Frequency of contact in (3) was divided a priori into categories per month (0, 1–5, 6–10, and > 10 contacts per month), per the SaniPath Exposure Assessment Tool [44, 46, 47]. The target respondent was the female head of household, as she was the primary caregiver and generally in charge of WASH in the household. GPS points were collected on Garmin eTrex Venture HC devices at the time of household survey and used to map locations of reported drain flooding, as a proxy for determining locations of routine drain flooding.

### Meteorological data (publicly-available)

Cumulative monthly rainfall data were obtained for Vellore district, Tamil Nadu state for January 2010 – December 2014 from the Customizable Rainfall Information System (CRIS), Hydromet Division of the India Meteorological Department website [45]. Monthly rainfall totals (in cm) were matched to the month of stool specimen collection. These data were then used, in combination with reported drain flooding, to estimate incidence of drain flooding.

Monthly average temperature data were obtained for Vellore district, Tamil Nadu state for January 2010 – December 2014 from World Weather Online [48]. Monthly average temperatures (in degrees Celsius) were matched to the month of stool specimen collection and included directly in the statistical models.

### Household socioeconomic status

Methods for collecting household socioeconomic status (SES) have been previously described [15]. Briefly, household SES was quantified at three or four time-points per household during the longitudinal MAL-ED study through construction of site-specific household asset indices and categories for household income and mother’s highest education, as previously described [52]. SES values were assigned to stool specimens temporally (all values from the household’s first assessment were assigned to all stool specimens collected from the child prior to the first assessment through the second assessment, all values from the second assessment were assigned to stool specimens collected between the second and third assessments, and so on). Although WASH indicators were originally included in the larger, composite “WAMI” index (water and sanitation, assets, mother’s education, and income), they were excluded from this analysis due to anticipated collinearity with household toilet and drain flooding data [52].

### Analyses

Maps were constructed in ArcMap version 10.1 (ESRI, Redlands, CA, USA). GPS points from household survey data were used to assess most-likely clustering of reported drain flooding in space using SaTScan version 9.4. In this software, Kulldorff’s Bernoulli spatial scan evaluates point data with binary values (e.g. flooded or not flooded) to assess the distribution of ‘0’ and ‘1’ values in space for non-random clustering [53]. Thus, we were able to assess whether and where spatial clustering of household-reported routine drain flooding was present, as an indication of areas where drains may routinely flood when cumulative monthly rainfall increases.

Modeling analyses were conducted in R version 3.1.2 (R Foundation for Statistical Computing, Vienna, Austria) using standard packages and the “lme4” package for mixed effects models [54]. Enteric infection, defined as detection of at least one enteric pathogen in a child’s routine or diarrheal stool sample, was the primary outcome. Mixed effects binomial regression models with a random effect for the child sampled were used to estimate associations between household or neighborhood flooding and enteric infection over the study period.

All multivariable regression models testing the effects of flooding were adjusted for household sanitation, total monthly rainfall (in cm), monthly average temperature, type of stool collected (routine or diarrheal), and household SES (asset index, income, and mother’s education). The flooding-related main variables of interest compared in multivariable models were the following:

1. Reported drain flooding, measured as a) any vs. no reported drain flooding from individual household surveys, and b) by comparing households inside a spatial cluster of reported drain flooding (e.g. low-lying area with drain flooding) vs. those outside of the spatial cluster;
2. Reported house flooding (similar comparison groups as for reported drain flooding);
3. Reported frequency of contact with drain or flood water (aspatial frequencies of children’s reported exposures to drain or flood waters were modeled as: a) any vs. no contact; and b) highest frequency of contact vs. no contact.).

Interactions of each of these main effects with monthly rainfall were tested as an estimation of the incidence of drain flooding (on a monthly scale/resolution) and were included in the model if significant. For example, a variable indicating a household or spatial cluster with reported routine drain flooding was interacted with a variable of monthly cumulative rainfall. A significant interaction would subsequently indicate that the effect of a child living in that household or spatial cluster with reported routine drain flooding on their odds of enteric infection is modified by rainfall (i.e. if it increases with rainfall, that may suggest rainfall-induced flooding affects odds of enteric infection). An α of 0.05 was used for all tests of significance.

## Results

### Household and neighborhood environments, exposure behaviors, and rainfall in the study neighborhood

Characteristics of the household and neighborhood environments and the child’s exposure-related behaviors were quantified through surveys with the adult caregiver, while monthly rainfall was quantified from the Indian Meteorological Department [45] (Table 1). Few households reported having toilets (33%). All household toilets were pour-flush. Most household toilets (82%) discharged directly into an open drain, while only 3 (9%) contained excreta onsite in a septic tank. Open drains were common near households, and many respondents with drains in front of their household (58%) reported that the drain would flood when it rained. While more than 80% of respondents reported that their children had monthly contact with drains and floodwater, frequent contact (> 10 contacts per month) with drains or floodwater was limited (15 and 26%, respectively). Rainfall was variable throughout the year, and heaviest during the southwest and northeast monsoons.

**Table 1:** Reported household/neighborhood conditions, exposure behaviors, and rainfall, 2010–2014

Household conditions^a^	Number (%)
Reported household toilet	33 (33.0)
Direct discharge to drain	27 (81.8)
Excreta contained onsite	3 (9.1)
Other/Don’t know	3 (9.1)
Neighborhood conditions^a^	
Open drain in front of household	96 (96.0)
*Flooding*	
Drain floods	57 (57.6)
House floods	23 (23.0)
Reported exposure behaviors^a,b^	
*Drain contact*	
Any	86 (86.0)
> 10 times per month	15 (15.0)
*Flood water contact*	
Any	82 (82.0)
> 10 times per month	26 (26.0)
Monthly rainfall (cm)^c^	Mean (SD)
Year-round	7.1 (6.0)
Dry season (January–May)	2.9 (3.5)
Southwest monsoon (June–September)	10.8 (4.2)
Northeast monsoon (October–December)	10.3 (6.3)

^a^Data from household survey (*n* = 100 households); ^b^The exposure behavior represents that of the study child, as reported by the adult respondent; ^c^Data from the India Meteorological Department [45]

**Fig. 1:**
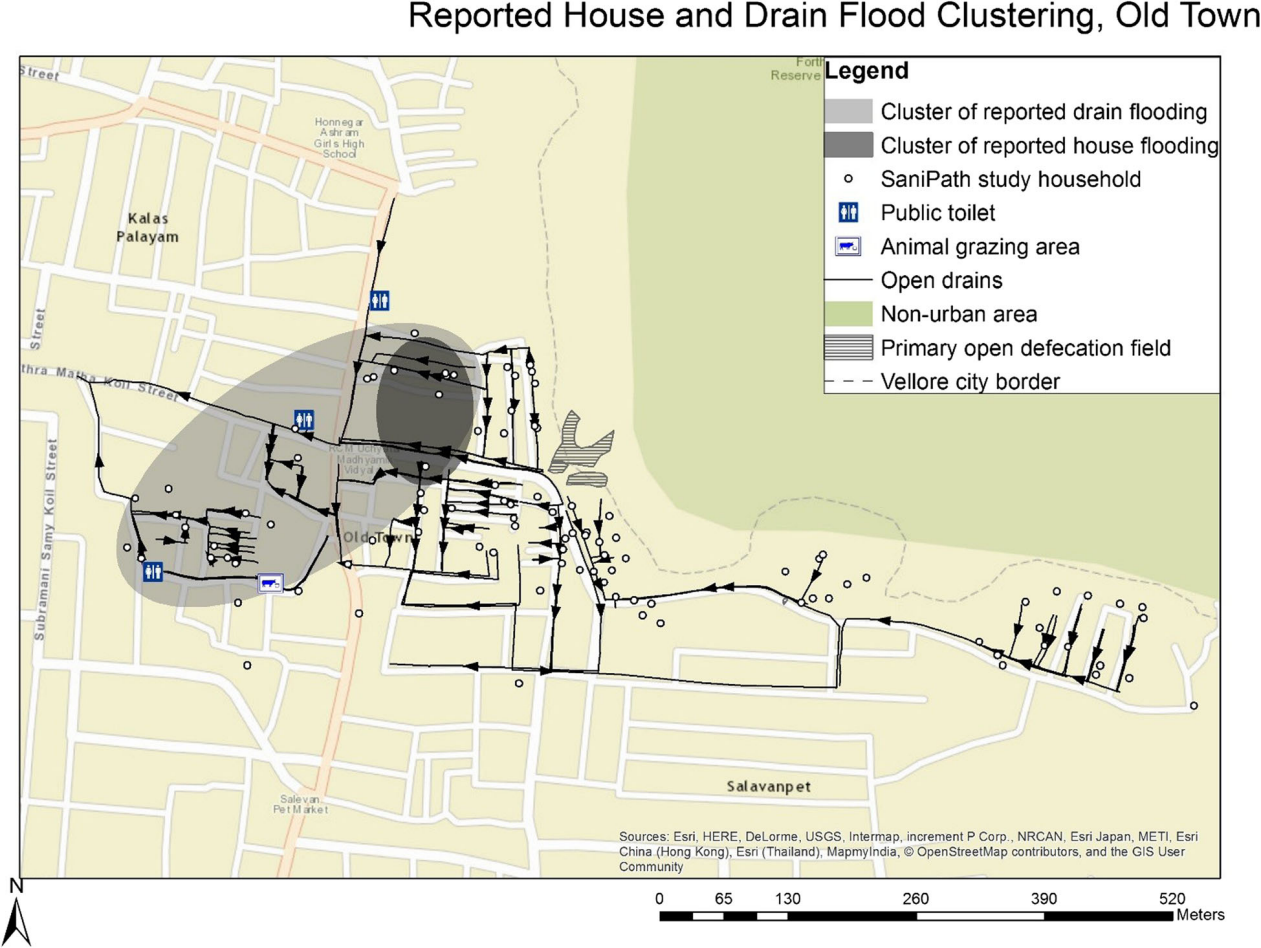
Reported drain and house flood clustering, Old Town*. Significant clusters* of reported drain and house flooding, determined by Kulldorff’s Bernoulli spatial scan [53], are presented using light and dark gray ellipses, respectively. Each SaniPath study household (represented by white dots) within each of these clusters reported flooding. Black lines represent drains, with arrows indicating the direction of drain flow. Only drains within the Old Town neighborhood boundary are presented. Base map:©OpenStreetMap contributors, data is available under the Open Database License, cartography is licensed as CC BY-SA (www.openstreetmap.org/copyright)

Potential fecal exposure points and neighborhood drains were mapped with significant most-likely clusters of reported flooding (determined by Kulldorff’s Bernoulli spatial scan, Fig. 1). Neighborhood drains flowed from east/northeast to west/southwest. One significant cluster of drain flooding (24 households) and one significant cluster of house flooding (7 households) were detected. Both clusters were downstream of the open defecation field, one of the highest elevation points in the neighborhood. Within each cluster, flooding was reported in 100% of the study households.

### Detection of pathogenic organisms in children’s stool

Detection of enteropathogens in stool specimens has been described previously [15]. Briefly, 3,754 specimens were collected from 230 children in Vellore during the MAL-ED study, 1,650 of these stool specimens were collected from children in SaniPath study households. Approximately 67% of routine and 80% of diarrhea stool specimens had at least one pathogen detected. Bacterial pathogens were most commonly observed in the population, with low to moderate occurrence of coinfections (about 2–12% of stool collected per child). The prevalence of enteropathogens in stool from children in the SaniPath study households did not differ significantly from that of children in all the MAL-ED study households in the Vellore study site.

### Unadjusted associations between household and neighborhood characteristics and enteric infection

Unadjusted associations between household or neighborhood characteristics and enteric infection were tested by mixed effects logistic regression for all stool specimens from SaniPath study households (Table 2). Odds of enteric infection were significantly lower for children in households that reported having a toilet (26% lower, OR: 0.74, 95% CI: 0.56–1.00) and increased significantly with increasing income category (OR: 1.09, 95% CI: 1.00–1.18) and monthly average temperature (OR: 1.05, 95% CI: 1.02–1.09). There was no significant variation in odds of enteric infection with cumulative monthly rainfall, reported drain flooding, or reported house flooding. Compared to households that reported their children had no monthly contact with drain water, the odds of enteric infection were 84% higher for children in households that reported that their child had frequent contact (more than 10 contacts per month) with open drains (OR: 1.84, 95% CI: 1.04–3.25). Compared to households that reported their children had no monthly contact with flood water, odds of enteric infection were 43% higher for children in households that reported that their child had any monthly contact with flood water (OR: 1.43, 95% CI: 0.99–2.06), though the finding was not statistically significant. Odds of detecting an enteric pathogen were 59% lower in asymptomatic stool compared with diarrheal stool specimens (OR: 0.41, 95% CI: 0.29–0.58).

**Table 2:** Unadjusted relationships between household and neighborhood conditions and pathogen detection in stool collected from children in SaniPath households, 2010–2014

a) Household conditions	Enteric infection Unadjusted OR (95% CI)
Household toilet	0.74 (0.56, 1.00)^†^
Household asset index (0–8)	0.99 (0.93, 1.06)
Household income category (0–8)	1.09 (1.00, 1.18)^†^
Mother’s education category (0–8)	0.95 (0.88, 1.03)
b) Neighborhood conditions	
Monthly total rainfall (cm)	1.00 (0.98, 1.02)
Monthly average temperature (°C)	1.05 (1.02, 1.09)^††^
*Flooding*	
Drain flooding	1.23 (0.93, 1.64)
House flooding	1.01 (0.72, 1.41)
No reported contact with drain water	Ref.
Any reported contact with drain water	1.50 (0.97, 2.32)
High (>10x/month) reported contact with drain water	1.84 (1.04, 3.25)^†^
No reported contact with flood water	Ref.
Any reported contact with flood water	1.43 (0.99, 2.06)
High (>10x/month) reported contact with flood water	1.19 (0.74, 1.91)
c) Stool collection	
Monthly (asymptomatic stool) collected^a^	0.41 (0.29, 0.58)^†††^

^a^compared to stool collected during diarrheal episodes; ^†^*p* < 0.05, ^††^*p* < 0.01; ^†††^*p* < 0.001

### Multivariable associations between neighborhood flooding and enteric infection

Associations between reported locations of routine drain and house flooding, rainfall, and enteric infection were evaluated in multivariable models, controlling for household sanitation, type of stool collected (routine vs. diarrheal), household asset index, income, average monthly temperature, and mother’s education. Interactions of monthly rainfall totals and locations of flooding (individual or clusters of households reporting flooding) were tested in each model and included if significant (Table 3a). Odds of enteric infection for children in households that reported drain flooding increased significantly with monthly rainfall (4% increased odds per cm of rainfall, OR: 1.04, 95% CI: 1.00–1.07). Children in the significant cluster of households that reported drain flooding also had significantly higher odds of enteric infection with increasing monthly rainfall (5% increased odds per cm of rainfall, OR: 1.05, 95% CI: 1.01–1.09). Average rainfall during the monsoon season was ≥10 cm per month, corresponding to a 40–50% increased odds of enteric infection, on average, during those months. Children in individual households or in the cluster of households that reported house flooding did not have significantly increased odds of enteric infection.

**Table 3:** Multivariable relationships between flooding in neighborhood, rainfall, and enteric pathogens detected in stool collected from children in SaniPath households, 2010-2014^a^

a) Flooding of neighborhood and household infrastructure	Enteric infection Adjusted OR^a^ (95% CI)
Reported drain flooding	0.97 (0.66, 1.42)
Monthly total rainfall (cm)	0.98 (0.95, 1.00)
Reported drain flooding x Monthly total rainfall (cm)	1.04 (1.00, 1.07)^†^
Cluster of reported drain flooding	0.72 (0.46, 1.11)
Monthly total rainfall (cm)	0.99 (0.97, 1.01)
Cluster of reported drain flooding x Monthly total rainfall (cm)	1.05 (1.01, 1.09)^†^
Reported house flooding	1.02 (0.73, 1.43)
Monthly total rainfall (cm)	1.00 (0.98, 1.02)
Reported house flooding x Monthly total rainfall (cm)	NS
Cluster of reported house flooding	1.05 (0.60, 1.83)
Monthly total rainfall (cm)	1.00 (0.98, 1.02)
Cluster of reported house flooding x Monthly total rainfall (cm)	NS
b) Reported contact	
Any reported contact with drain water	1.36 (0.88, 2.08)
Monthly total rainfall (cm)	1.00 (0.98, 1.02)
Any reported contact x Monthly total rainfall (cm)	NS
High (>10x/month) reported contact with drain water	1.28 (0.68, 2.38)
Monthly total rainfall (cm)	0.98 (0.94, 1.01)
High reported contact x Monthly total rainfall (cm)	NS
Any reported contact with flood water	1.42 (1.00, 2.01)
Monthly total rainfall (cm)	1.00 (0.98, 1.02)
Any reported contact x Monthly total rainfall (cm)	NS
High (>10x/month) reported contact with flood water	1.28 (0.80, 2.06)
Monthly total rainfall (cm)	1.02 (0.99, 1.05)
High reported contact x Monthly total rainfall (cm)	NS

^a^In addition to covariates shown, all models were adjusted for type of stool collected, monthly average temperature, presence/absence of household sanitation, household asset index, household income, and mother’s education. Model ORs for all covariates are available in the Supplementary Material. “NS” indicates interaction of rainfall and main effect was not significant at 0.05 and thus not included in model; ^†^*p* < 0.05

### Multivariable associations between reported contact with neighborhood drain and flood water and enteric infection

Associations between reported frequencies of contact with drain or flood water, rainfall, and enteric infection were evaluated by multivariable, mixed effects logistic regression, controlling for type of stool collected (routine vs. diarrheal), household sanitation, household asset index, household income, and mother’s education. Interactions with reported frequency of contact and rainfall were tested in each model and included if significant (Table 3b). Though all the ORs for the main effects were greater than 1.0, the associations between reported frequencies of monthly contact with drain water or flood water and enteric infection were not statistically significant.

## Discussion

The goal of this study was to evaluate the associations between open drains, areas of reported flooding, and pediatric enteric infection in a low-income, urban setting where fecal sludge management was poor and open drains were common. Children in individual households that reported that drains flooded, as well as those in the cluster of households reporting drain flooding (which were located downstream on the drainage system), had significantly increased odds of enteric infection as cumulative monthly rainfall increased when compared to children in areas without reported flooding. These results suggest that rainfall-induced open drain flooding may be associated with pediatric enteric infection in this setting. Specifically, estimates of 4–5% increased odds of enteric infection per cm of monthly rainfall in these models suggest that these children had at least 40–50% higher odds of enteric infection, on average, during the monsoon seasons compared with children in the rest of the neighborhood. Enteric infection was not significantly associated with reported flooding of households specifically, or frequency of contact with flood or drain water and rainfall, when adjusting for household sanitation, SES, and average monthly temperature. The absence of a significant association with frequency of flood or drain water contact may be due to pathogen loads in drains being high enough to cause infection with a single exposure. Taken together, these results suggest that pediatric enteric infections were associated with drain flooding due to neighborhood geography, and not behavioral factors (i.e. were not elevated in a dose-response manner with the reported frequency of child contact with drain or flood water). These findings highlight the risks associated with the spread of fecal contamination in public environments due to poor neighborhood FSM, drainage, rainfall, and flooding.

This study is the first to examine interactions between FSM in the urban public environment (including open drains and flooding), rainfall, and enteric infection in a pediatric cohort that captured most enteric infections during the first two years of life. Urban studies of environmental transmission pathways of infection, and particularly knowledge of risks associated with open drains, have been primarily limited to modeling risks from specific types of pathogens using QMRAs [9, 10, 13]. While these studies identified open drains as a risk factor for enteric infections, their models have suggested a dose-response relationship between frequency of contact with drains and pediatric enteric infections [9, 10, 13]. In contrast, we observed evidence of increasing odds of enteric infection with drain flooding and neighborhood geography, modified by rainfall but not frequency of contact, in this study.

We hypothesize that poor containment of feces along the entire sanitation chain contributed to neighborhood-level environmental contamination in drains, which in turn could yield increases in enteric infection in children living in areas with reported drain flooding when monthly rainfall was high. Given the low observed coverage of toilets with onsite containment, the absence of affordable and reliable emptying services for these facilities [unpublished data], and the absence of sewage treatment facilities [unpublished data], the open drains in this neighborhood acted as de facto sewerage and solid waste disposal systems [47]. These drains received excreta directly from household toilets and open defecation, as well as indirectly from run-off from the high elevation open defecation field. Both of these fecal sources may have resulted in concentrations of fecal contamination high enough to cause infection after a single contact with an open drain [9–12, 28, 37, 40, 41, 55]. A cross-sectional study of Old Town and a nearby neighborhood showed that open drains in clusters of household toilets that discharged directly to open drains had higher concentrations of human-specific fecal contamination (2.50 log_10_ genome copies higher, as measured by GII norovirus) than elsewhere in the study area [28]. These drains could have been easily blocked by solid waste or feces and flooded in months with high rainfall, given their open construction and small size (diameter and depth) near to the household [56]. The close proximity of open drains to most households suggests that study children could have had direct contact with fecal contamination in drain water or indirect contact through their parents’ or siblings’ exposures [57–59]. Thus, the proximity of open drains themselves—and concentrations of pathogens in open drains—were likely sufficient to significantly increase the odds of enteric infection as rainfall increased. This result may explain why odds of infection for young children did not additionally increase with increased intensity of flooding (measured by flooding of the house, as opposed to only the drains themselves) or with increasing frequency of drain contacts. For example, children in households that did not flood (but whose drains nevertheless flooded) may have received sufficient exposures from that drain flooding alone to cause infection. Additionally, the absence of a dose-response relationship between drain contact and enteric infection may be explained by a single exposure having a dose of enteric pathogens that is sufficient to cause infection. Thus, in contrast to previous QMRAs [9, 10, 37], the elevated odds of infection in children in this cohort was based on the location of their household alone, and did not increase with the frequency of children’s contact with open drains in a dose-response manner.

The absence of such a dose-response relationship suggests that children in households, and their parents, would be unable to mitigate their exposures and subsequent odds of infection by themselves. For example, reducing contact with open drains through individual behaviors would be ineffective if the dose in a single exposure is sufficient to cause infection. Therefore, common infrastructure solutions that reduce, but do not eliminate, contact with drains (such as fences or drain covers) would also be ineffective if drains routinely overflow their bounds [9, 10]. Thus, neighborhood-level interventions to improve FSM and reduce flooding are necessary to mitigate exposure to fecal contamination and reduce pediatric enteric infection burdens in the community.

Measures of household assets, income, and mother’s education from robust, multiple-time-point assessments during the MAL-ED follow-up period minimized confounding due to SES within the study population [52]. Although the observation of increased unadjusted odds of enteric infection with increasing income is not consistent with well-established links between SES and improved child health [60], the assets, mother’s education, and income sub-components of the WAMI index [52] should be not be viewed individually because each alone is not necessarily a good indicator of household socioeconomic status.

This study has some other notable strengths and limitations in outcome and exposure measurements. Measurement of enteric infection from stool specimens collected regularly over the first two years of life provided a sensitive and objective health outcome measure for examining associations with environmental exposures compared to use of self-reported diarrhea alone [4–6, 61, 62]. However, exposure measurements were collected when the children were 2–5 years old, while infection outcomes were measured during the first two years of life, which necessitated static assumptions for household and environmental conditions and behaviors and may have overestimated exposure frequencies [59]. Sensitivity analyses from a previous study in this neighborhood suggest that there was little change in household sanitation infrastructure over the study period [15], though neighborhood-level FSM infrastructure like drain construction was not measured. Because these findings were primarily based on the location of drains and drain-flooding, which were unlikely to change over time given that drains were mapped at the start of the study period and locations confirmed at the end of it, we do not feel the validity of our findings is threatened. However, because direct observation and measurement of flooding was not feasible, we approximated drain flooding from a combination of household-reported locations of routine flooding and district-level meteorological data collected monthly. This approach, combined with the aggregation of enteric infection data to month-level, may have reduced the precision of the estimates of the magnitude and geographic scope of neighborhood flooding and limited our ability to account for temporal lags that may influence rainfall-enteric infection relationships [39, 45]. Future studies should track exact geographic and temporal scales of flooding to provide more precise estimates of associations with health outcomes.

Mitigation of pediatric enteric infection in urban environments requires understanding neighborhood-level infrastructure and geography, magnitude of environmental contamination, and frequency of behaviors associated with exposure to fecal contamination. Household-level WASH interventions alone may not be sufficient to reduce these risks [63]. Household-level sanitation interventions must be complemented by neighborhood-level FSM efforts to contain feces along the entire sanitation chain and reduce fecal contamination in the environment. Future studies should contextualize the effects of rainfall and flooding on enteric infection with neighborhood-level FSM, and consider the relationships between local infrastructure, rainfall, and behaviors. Studies that examine public domain interventions and associated health effects in the context of the contributions of behavioral, infrastructural, or geographic factors to fecal exposures may be more likely to identify specific intervention points [64].

## Conclusions

In this setting, significant associations between pediatric enteric infection and areas subject to flooding of open drains were observed due to inadequate local sanitation infrastructure (including neighborhood-level FSM) and heavy rainfall, independent of the reported frequency of contact with open drains. Because households are less likely to be able to prevent these exposures to fecal contamination from floods through changing individual behaviors alone, changes to local infrastructure must occur to mitigate these exposures within public environments. Efforts to design effective interventions to improve the health of children in low-income, urban neighborhoods need to recognize both the interconnectedness of the public and private domains and the associations between infrastructure, geography, rainfall, and exposure behaviors in the transmission of enteric pathogens.

## Additional file

Additional file 1: Organisms tested in stool specimens. (DOCX 13 kb)

## Data Availability

Datasets for analysis of the MAL-ED study data can be found by contacting the authors of the MAL-ED study [6, 43]. SaniPath data is available by contacting the Emory Center for Global Safe WASH (http://www.cgswash.org/).

## Abbreviations

CMC: Christian Medical College
FSM: fecal sludge management
IRB: Institutional Review Board
MAL-ED study: The Etiology, Risk Factors, and Interactions of Enteric Infections and Malnutrition and Consequences for Child Health and Development Project
OR: Odds ratio
QMRA: Quantitative microbial risk assessment
