# Supplementary material for: Associations between open drain flooding and pediatric enteric infections in the MAL-ED cohort in a low-income, urban neighborhood in Vellore, India
Source: BMC Public Health. 2019 Jul 10;19:926. doi: 10.1186/s12889-019-7268-1 (PMC6617624; doi:10.1186/s12889-019-7268-1)
Supplement: Supplementary file 1 — Organisms tested in stool specimens. (DOCX 13 kb) [file 12889_2019_7268_MOESM1_ESM.docx]

Supplemental information (SI):

*Organisms tested in stool specimens:*

All stool specimens collected from children in the Old Town neighborhood were tested for the following bacteria: *Salmonella*, *Shigella*, *Vibrio*, *Yersinia*, *Aeromonas*, *Plesiomonas*, diarrheagenic *E. coli* (Shiga toxin-producing *E. coli*, enterotoxigenic *E. coli*, enteropathogenic *E. coli*, enteroinvasive *E. coli*, and enteroaggregative *E. coli*), and *Campylobacter* spp. Protozoa tested included *Balantidium coli*, *Cryptosporidium*, *Chilomastix mesnili*, *Cyclospora*, *Entamoeba histolytica*, *Giardia lamblia*, *Endolimax nana*, *Iodamoeba butschlii*, and *Isospora*. Helminths included *Hymenolepis nana*, *Strongyloides stercoralis*, *Ascaris lumbricoides*, *Taenia* spp., *Trichuris trichiura*, *Schistosoma*, *Enterobius vermicularis*, *Hymenolepis diminuta*, and hookworm species. Finally, viruses included genogroup I and II norovirus, astrovirus, rotavirus, and adenovirus [2, 44].
